# Safety and effectiveness of seahorse extract (*Hippocampus comes* L.) on the hematological profile and body weight of male rats induced by depo medroxyprogesterone acetate

**DOI:** 10.5455/javar.2024.k822

**Published:** 2024-09-29

**Authors:** Trisnawati Mundijo, Franciscus Dhyanagiri Suyatna, Agung Eru Wibowo, Yusra Yusra, Yurnadi Hanafi Midoen

**Affiliations:** 1 Department of Medical Biology, Faculty of Medicine, Universitas Muhammadiyah Palembang, Palembang, Indonesia; 2 Department of Pharmacology and Therapeutics, Faculty of Medicine, Universitas Indonesia, Jakarta, Indonesia; 3 Research Centre for Pharmaceutical Ingredient and Traditional Medicine, National Research, and Innovation Agency, Serpong, Indonesia; 4 Department of Clinical Pathology, Faculty of Medicine, Universitas Indonesia, Jakarta, Indonesia; 5 Department of Medical Biology, Faculty of Medicine, Universitas Indonesia, Jakarta, Indonesia

**Keywords:** Body weight, DMPA, effectiveness, hematological profile, safety, seahorse

## Abstract

**Objective:**

The objective of this study was to investigate the effectiveness and safety of seahorse *(Hippocampus comes *L.) extract on hematological profile and body weight on rats induced by depo medroxyprogesterone acetate (DMPA).

**Materials and Methods:**

Thirty adult male Sprague-Dawley rats with 200–250 gm and 8 weeks old. All rats were intramuscularly administered 1.25 mg/kg BW DMPA Merck Depo Geston at 150 mg/3 ml. The animals were divided into five main groups (6 each), consisting of aquadest (G1), CMC 1% (G2), seahorse extract (SE) dose 150 mg/kg BW (G3), 225 mg/kg BW (G4), and 300 mg/kg BW (G5). All rats were weighed until the end of the treatment week.

**Results:**

The hematological profile and body weight of the group given SE tended to increase compared to the group not given extract; however, our hematological profile and body weight were in the normal range for rats.

**Conclusion:**

We find that SE enhances the effectiveness of the hematological profile, body weight, and safety of rats induced by DMPA.

## Introduction

The ocean covers about 70% of the earth’s surface. Hence, more than 90% of all living organisms are found in the marine. The marine has biodiversity as a natural product with bioactive compounds that are different from the organism nonmarine, which is an important source of leading compounds in the research and development of new drugs [1]. However, current research on marine organisms is still limited, one of which is seahorse.

Seahorse (*Hippocampus* spp.), a marine fish that has more value in humans, is an ethnopharmacological species such as fertility and antiapoptotic [2–4]. Indonesia is an archipelagic country with a high biodiversity [5], one of which is seahorses. Of the 48 types of seahorses in this world [6], one of which in Indonesia is *Hippocampus comes* L. [7]. Studies in Indonesia on this species are conducted on biocompounds [8]. This condition requires further exploration of the benefits of seahorse *H. comes* L. for human health as an exciting natural product potential.

To date, the World Health Organization (WHO) has focused on natural products, with WHO’s traditional medicine strategy conducting a comprehensive analysis of conventional medicine. Thus, the efficacy of using natural products should be evaluated, one of which is the hematological profile and body weight [9]. Nowadays, seahorse have increasingly been used in research to explore their benefits, especially to medical health, such as fertility. The earlier study stated that seahorses might enhance male reproductive dysfunction in rats [10]. The safety of substances in seahorses that have a role in human health must be ensured. One evaluation of the use of a substance is carried out through a hematological profile and body weight. Our research evaluates the safety level of seahorse extract (SE) as a fertility enhancer on hematological profile and body weight in experimental animals.

## Materials and Methods

### Ethical approval

Ethical approval by the ethics committee of the Faculty of Medicine, Universitas Indonesia, with protocol number KET-101/UN2.F1/ETIK/PPM.00.02/2021.

### Animals and treatment

The study is experimental with 30 adult male Sprague-Dawley (SD) rats (200–250 gm; 8 weeks old) from the Center for Drug and Food Control, Indonesia. The SE in this study is from our material collection [11]. The SD rats were acclimatized for 1 week and treated. They were also kept at Animal Research Facilities—Indonesian Medical Education and Research Institute (ARF-IMERI), Faculty of Medicine, Universitas Indonesia.

The SD rats were acclimatized for 1 week with 25°C, a 12 h light/dark cycle, and free access to food and water. The SD rats were randomly categorized into five main groups (6 rats each) consisting of aquadest (G1), CMC 1% (G2), SE dose 150 mg/kg BW (G3), 225 mg/kg BW (G4), and 300 mg/kg BW (G5). All animals were weighed every morning all day in treatment and intramuscularly administered 1.25 mg/kg BW DMPA Merck Depo Geston at 150 mg/3 ml in the right or left thigh at weeks 0 and 12. SE was administered for treatment from weeks 7 to 18. In last week’s experiment, all rats were euthanized with ketamine Ket-A-100 at 100 mg/kg BW and Xylazine Xyla Holland at 10 mg/kg BW for the rat.

### Assessment of hematological profile

After euthanasia, 2.5 ml of blood from the heart was at once taken with a syringe and transferred into a vacuum tube with ethylene diamine tetra-acetic acid (EDTA) to obtain whole blood. The whole blood was analyzed using flow cytometry for the parameters of erythrocytes, hemoglobin, hematocrit, platelets, leukocytes, mean corpuscular volum (MCV), mean corpuscular hemoglobin (MCH), mean corpuscular hemoglobin concentration (MCHC), and red blood cell distribution width (RDW) with the auto-hematology analyzer VABIO 580 Biota Group Saglik Sistemleri San Ve Tic Ltd Sti (China).

### Assessment of body weight

The rats were weighed every day until the 18th week using a weighing scale digital. It was carried out to determine whether the rat’s body weight changed during the treatment.

### Statistical analysis

The results were analyzed using the IBM SPSS statistics for Windows version. 20 and visualized with GraphPad Prism 9. All data are expressed as mean standard deviation (SD) and significance level that was accepted at *p *< 0.05.

## Results

**Figure 1.**
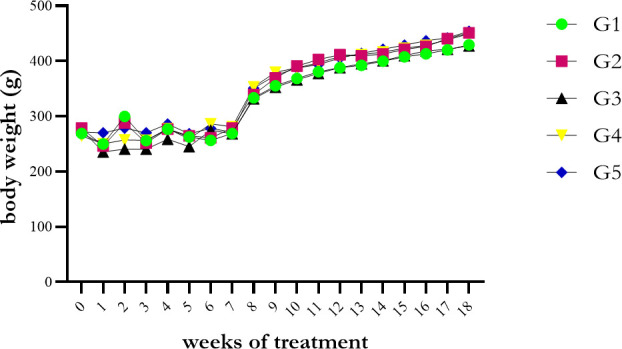
Body weight in rats. G1: Group with treatment aquadest, G2: CMC 1%. G3: SE dose 150 mg/kg BW, G4: SE dose 225 mg/kg BW, and G5: SE dose 300 mg/kg BW.

Our study observed hematological profile and body weight in rats after induced DMPA, shown in Table 1 and Figure 1. In Table 1, we saw that the highest level of erythrocytes and hemoglobin were found in G3, about 8.15 106/μl and 13.52 gm/dl. Hematocrit fraction was found in G5 at around 43.22%, and in G4, the highest of thrombocytes (861.8 × 103/μl), MCH (16.90 pg), MCHC (31.66 gm/dl), and RDW (15.88%). Parameters leukocyte and MCV, we found the highest in G2, about 8.00 × 103/μl and 54.30 fl.

**Table 1.** Hematological profile in rats.

Parameter (Mean ± SD)	G1	G2	G3	G4	G5	Normal range	*p-*value
Erythrocytes (10^6^/μl)	7.48 ± 1.96	7.70 ± 0.46	8.15 ± 0.37	7.83 ± 0.11	8.08 ± 0.66	3.88–9.99	0.618
Hemoglobin (gm/dl)	12.30 ± 3.23	12.18 ± 1.79	13.52 ± 0.84	13.30 ± 0.25	13.50 ± 1.17	10.4–17.6	0.637
Hematocrit (%)	40.24 ± 9.99	41.94 ± 3.33	42.82 ± 2.48	42.00 ± 0.63	43.22 ± 3.58	38.5–52.5	0.962
Thrombocyte’s (10^3^/μl)	575.6 ± 269	678.4 ± 215	756.8 ± 123.8	861.8 ± 306	817.0 ± 308.7	574.0–1,253.0	0.08
Leukocytes (10^3^/μl)	6.55 ± 1.4	8.00 ± 2.1	6.20 ± 1.4	7.85 ± 2.5	6.22 ± 2.0	1.98–11.1	0.40
MCV (fl)	54.06 ± 2.21	54.30 ± 1.51	52.68 ± 1.57	53.58 ± 1.18	53.48 ± 0.83	46.3–56.2	0.522
MCH (pg)	16.50 ± 0.46	17.12 ± 0.50	16.60 ± 0.59	16.96 ± 0.36	16.70 ± 0.50	16.3–19.5	0.353
MCHC (gm/dl)	30.50 ± 0.77	31.50 ± 0.40	31.58 ± 0.53	31.66 ± 0.30	31.26 ± 0.59	31.9–38.5	0.019
RDW (%)	15.70 ± 0.88	15.66 ± 0.71	15.04 ± 0.18	15.88 ± 0.58	15.80 ± 0.41	11.6–16.2	0.201

G1: Group with treatment aquadest, G2: CMC 1%, G3: seahorse extract dose 150 mg/kg BW, G4: seahorse extract dose 225 mg/kg BW, and G5: seahorse extract dose 300 mg/kg BW.

Indeed, it is, our results obtained in the parameter of the hematological profile were all in the normal range and were not statistically significant (*p* > 0.05), except in the MCHC parameter (*p* = 0.019; <0.05). In our present study, we found that the body weight has a fluctuating increase from each group up to 18 weeks of treatment. Results are shown in Figure 1. The average body weight increased after therapy for the SE, but not significantly for the rats between groups during the study.

## Discussion

Our study reported that the hematological profile in the group given SE tended to be higher than the other group. These facts show that the substance content in SE increases the hematological profile, nevertheless in the normal range. The seahorse *H. comes* L. extract has amino acids, indicated steroids, triterpenoids, and alkaloids [11]. These compounds affect the self-renewal and differentiation in hematopoietic stem cells and restrict progenitors in the blood system [12,13]. In addition, another study states that seahorse contains peptides and amino acids, which play a role in hematopoietic [12–15].

However, the parameter leukocytes obtained different results, and the highest was found in the group with DMPA induction and without SE. This result indicates that DMPA induction can cause suppression of GnRH. DMPA has several mechanisms, primarily inhibiting gonadotropin secretion [16]. This condition has a few contraindications that can suppress peripheral dendritic cells, T cells, and cytokines that can promote infections [17,18]. Chandra et al. [18] stated that exposure to a single dose of DMPA causes changes in the immune cell population, including an increase in leukocytes, although not statistically significantly different. The other study, reported by Govender et al. [19] mentions that DMPA use as a contraceptive may modulate immune function, inflammation, and cytokine expression changes. Studies in human and animal models stated that DMPA increases immune cells and affects the cellular and molecular levels, including immunosuppression of human T cells [20–22].

Progestin, one of DMPA’s components, can change gene expression after activating intracellular steroid receptors [23]. DMPA has changed the immune system, significantly decreasing interleukin-6 and interleukin-1β [24]. DMPA can increase inflammation and decrease immune activation [25]. During the experiment, the group given SE had the least number of leukocytes, which indicates that the compounds in SE have an anti-inflammatory effect, suppressing contraindications due to DMPA induction. Biocompounds in SE are reported to have an anti-inflammatory effect [26]. However, our study’s leukocyte fluctuations in rats were still within the normal range [27].

In another part of our result, we observed that SE enhanced thrombocyte levels. We observed that thrombocytes had significantly increased in the G4 and G5 groups (1.5 103/μl). In this condition, SE has a potential natural product that is helpful for thrombocytopenia but still needs further research.

Our study found that the MCHC parameter is significant from others. We found that G1 was the lowest and G4 the highest group. The MCHC shows the amount of hemoglobin per unit volume, calculated as MCH divided by MCV [28]. These facts align with our study in parameter MCH, which is G1 with the lowest group from another.

Our present study found that the hematological profile improved within the normal range by administrating sea horse extract. This fact confirms that sea horse extract influences hematopoiesis. Hematopoiesis is the cellular and cell-derived components of the blood [29], which is the erythropoiesis metabolism [30]. Erythrocytes affect the Hb, MCV, MCH, and MCHC levels. The total and size of erythrocytes influence hematocrite, while MCV is the average size of erythrocytes. MCH is the average hemoglobin count in red blood cells, while MCHC is the hemoglobin level compared to the hematocrite level. The hemoglobin, hematocrit, MCV, MCH, MCHC, and RDW are reported in the complete blood count. MCV is the average volume of red blood cells. Hematocrit is the percentage of packed red blood cells in the whole blood, MCH is the average amount of hemoglobin in the red blood cells, and MCHC is the average hemoglobin concentration in red blood cells. In contrast, RDW measures the degree of variation in red blood cells [31].

The authors found that rats’ body weight increased compared to before treatment. In the first week of treatment, the average body weight was decreased—this condition is about the acclimatization process. However, as of the study, the average body weight was in the normal range for adult male rats, from 300 to 500 gm [32]. Indeed, there was no significant difference in body weight in rats. Thus, it concluded that SE does not affect the rat’s body weight.

Our research conducted on experimental animals found that SE has the potential to be a candidate for a natural product that can increase fertility but not affect the hematological profile and body weight. It must be explored further; SE tends to be safely used as a marine natural product.

## Conclusion

The authors found that SE enhances the effectiveness of hematological profile and body weight, as well as safety in rats-induced DMPA.

## List of Abbreviations

ARF-IMERI, Animal Research Facilities-Indonesian Medical Education and Research Institute; DMPA, Depo medroxyprogesterone acetate; EDTA, Ethylene diamine tetra-acetic acid; MCH, Mean corpuscular hemoglobin; MCHC, Mean corpuscular hemoglobin concentration; MCV, Mean corpuscular volum; RDW, Red blood cell distribution width; SD, Sprague-Dawley; SD, Standard deviation; WHO, World Health Organization.
